# Crusted (Norwegian) scabies as a strong marker of adult T‐cell leukemia/lymphoma in HTLV‐1 infection

**DOI:** 10.1002/ccr3.1983

**Published:** 2019-01-31

**Authors:** César Bimbi, Piotr Brzezinski, Malgorzata Sokolowska‐Wojdylo

**Affiliations:** ^1^ Dermatologia Medica & Laser Clinic Porto Alegre Brazil; ^2^ Brazilian Society of Dermatology Porto Alegre Brazil; ^3^ Department of Dermatology 6th Military Support Unit Ustka Poland; ^4^ Department of Dermatology Provincial Specialist Hospital in Slupsk Ustka Poland; ^5^ Institute of Biology and Environmental Protection Pomeranian Academy Slupsk Poland; ^6^ Department of Dermatology, Venereology and Allergology Medical University of Gdansk Gdansk Poland

**Keywords:** adult T‐cell leukemia/lymphoma, crusted scabies, human T‐cell lymphotropic virus 1, predictive marker

## Abstract

We present a rare case of crusted scabies in an human T‐cell lymphotropic virus type 1 (HTLV‐1) infected woman prior to onset of adult T‐cell leukemia/lymphoma (ATL). We highlight the importance of this rare form of scabies as a prediagnostic sign of ATL, requiring high suspicion and monitoring of possible symptoms for early detection of ATL.

## 1 INTRODUCTION

Crusted (Norwegian) scabies is strongly associated with human T‐cell lymphotropic virus type 1 (HTLV‐1) infection.^1^ We report a case of crusted scabies (CS) that occurred in an HTLV‐1 infected patient prior to the onset of adult T‐cell leukemia/lymphoma (ATL).

## 2 CASE REPORT

**Figure 1:**
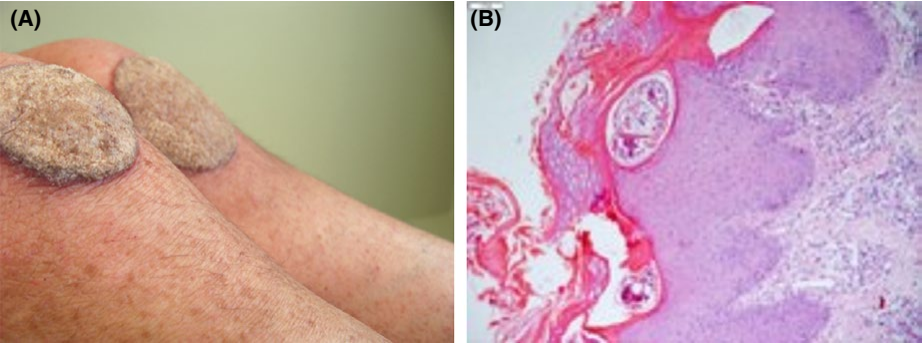
Crusted scabies presenting as plaque lesions affecting the knees, simulating psoriasis (A). Histopathology shows parakeratosis, acanthosis, and burrows containing mites in the stratum corneum (B)

**Figure 2:**
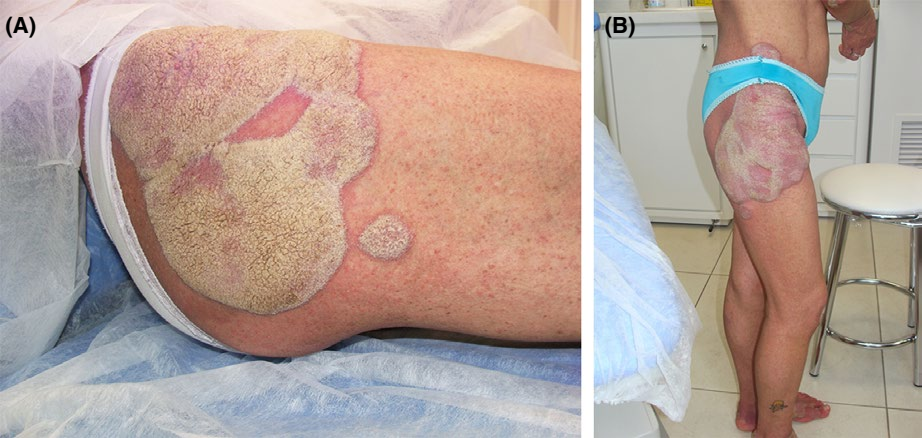
Extensive coriaceous plaques around the pelvis elevated about 3 mm above the skin surface (A). Note the punctiform vesicles disseminated on the lower limbs (B)

**Figure 3:**
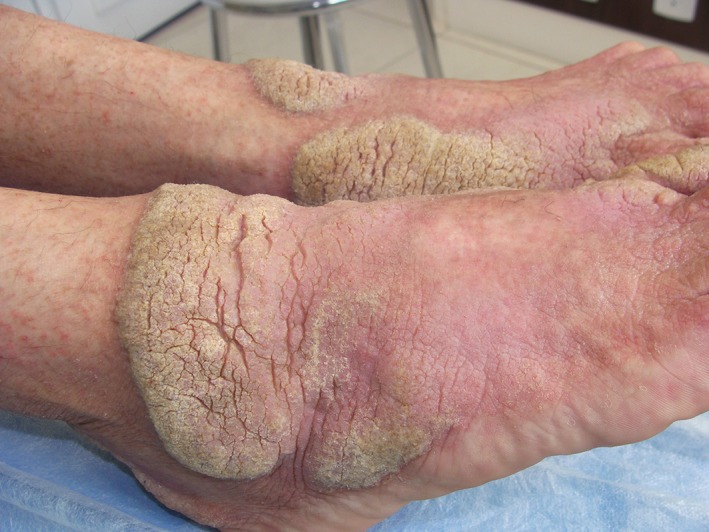
Elevated verrucous vegetating plaques on the feet

A 41‐year‐old woman presented with unusual extensive plaque lesions covered with thick, hyperkeratotic, sharp‐edged, silvery‐white scales symmetrically distributed on the knees (Figure 1A), pelvic girdle (Figure 2A,B), and feet (Figure 3). Vesicles were disseminated on skin areas not covered by the plaques. She reported that the lesions had appeared 1 year earlier, and she received topical corticosteroids for a diagnosis of psoriasis. Repeated HIV tests were negative. A review of her medical records revealed a diagnosis of hepatitis C and cervical ganglion tuberculosis before the appearance of skin lesions. Both conditions were treated, and the patient was discharged in good condition.

A skin biopsy revealed *Sarcoptes scabiei* mites in burrows in the stratum corneum (Figure 1B). Treatment was started with topical permethrin 5% daily for 7 days, and then twice weekly for 2 weeks plus oral ivermectin (200 μg/kg/dose) given on days 1, 2, 8, 9, and 15. The skin lesions cleared rapidly.

Seven months after complete clearance of crusted lesions, her general health suddenly worsened with intermittent fever, severe weight loss of 10 kg, enlarged lymph nodes, fatigue, night sweating, malaise, and bruised skin. She was admitted to the Hematology Unit and investigation revealed anemia, leukocytosis, hypercalcemia, and elevated lactate dehydrogenase. Samples were positive for anti‐HTLV‐1 antibodies as detected by enzyme‐linked immunosorbent assay (ELISA), confirmed via polymerase chain reaction (PCR). Further hematological investigation was carried out with bone marrow biopsy and computed tomography scan, showing intraabdominal lymphadenopathy that led to the diagnosis of ATL associated with HTLV‐1 infection. Hepatitis C also recurred.

A recurrence of crusted skin lesions was observed, this time on the scalp and ears. She received antiviral therapy with zidovudine, acyclovir, and alpha‐interferon against HTLV‐1. Subsequently, cycles of cyclophosphamide, doxorubicin, vincristine, and prednisone (CHOP) chemotherapy considerably improved the symptoms. Skin lesions cleared again with topical permethrin 5% plus oral ivermectin. One year after treatment, the patient is in good health.

## 3 DISCUSSION

Brazil has one of the highest prevalence rates of scabies worldwide. Regarding HTLV‐1, 2.5 million Brazilians are estimated to be infected.^2^

Crusted scabies is strongly associated with HTLV‐1 virus, and recent studies show that this infestation is also a predictive marker for ATL. In a study in French Guiana, an HTLV‐1 endemic area, four of six HTLV‐1 seropositive patients had concomitant ATL when CS was diagnosed, or developed ATL a few months later, suggesting that the occurrence of CS in these patients is a sign of ATL‐related immunosuppression^3^ or a prediagnostic sign of ATL.^4^

Human T‐cell lymphotropic virus was discovered only in 1980, when it was isolated from a patient with lymphoma^5^, ^6^; thus, it remains a topic of research interest. Knowledge of the pathogenesis is also recent, and a variety of symptoms and diseases potentially associated with HTLV have not yet been incorporated into the practice of medical investigation. These diseases and clinical manifestations include HTLV‐associated myelopathy, spastic paraparesis, urinary incontinence, erectile dysfunction, lumbar pain, lower limb sensitivity, intestinal constipation, infectious inflammatory diseases (especially of the lungs), polymyositis, fibromyalgia, and the dry syndrome, with lacrimal and salivary gland involvement.^2^, ^7^, ^8^ In tropical countries, *Strongyloides stercoralis* intestinal hyperinfection, or its skin form of larva currens (migrans), is common and always localized; therefore, when disseminated, it may be associated with HTLV‐1.^9^

For dermatologists, a chronic, unresponsive, recurrent, severe, exudative, foul‐smelling atopic eczema with crusting should raise suspicion. HTLV‐1 tests are required in resistant forms of childhood eczema. In cases of unresponsive seborrheic dermatitis with chronic nasal secretion and presence of opportunistic infections,^10^ serological tests should also be ordered.

This case supports previous but scarce reports in the literature demonstrating this rare form of scabies as a prediagnostic sign of ATL, requiring a high degree of suspicion of HTLV coinfection and careful monitoring of possible symptoms in order to detect the early development of ATL.

## CONFLICT OF INTEREST

None declared.

## AUTHOR CONTRIBUTIONS

CB is the principal investigator, provided medical care, made the diagnosis and treated the patient. This author conceived the study and takes overall responsibility for the integrity of the content of the manuscript. PB conducted a critical review of the literature, critically revised the manuscript, and supervised the study. MSW assisted in the design of the study, conducted a literature search and wrote the first draft of the report, revised the manuscript for important intellectual content and wrote the discussion. All authors read and approved the final manuscript.

## References

[1] Brites C , Weyll M , Pedroso C , Badaro R . Severe and Norwegian scabies are strongly associated with retroviral (HIV‐1/HTLV‐1) infection in Bahia, Brazil. AIDS. 2002;16:1292‐1293. 12045498 10.1097/00002030-200206140-00015

[2] Romanelli LC , Caramelli P , Proietti AB . Human T cell lymphotropic virus (HTLV‐1): when to suspect infection? Rev Assoc Med Bras. 1992;2010(56):340‐347. 10.1590/s0104-42302010000300021 20676544

[3] del Giudice P , Sainte Marie D , Gerard Y , Couppie P , Pradinaud R . Is crusted (Norwegian) scabies a marker of adult T cell leukemia/lymphoma in human T lymphotropic virus type I‐seropositive patients? J Infect Dis. 1997;176:1090‐1092. 9333174 10.1086/516518

[4] Murphy EL , Hanchard B , Figueroa JP , et al. Modelling the risk of adult T‐cell leukemia/lymphoma in persons infected with human T‐lymphotropic virus type I. Int J Cancer. 1989;43:250‐253. 2917802 10.1002/ijc.2910430214

[5] McLaughlin‐Drubin ME , Munger K . Viruses associated with human cancer. Biochim Biophys Acta. 2008;1782:127‐150. 18201576 10.1016/j.bbadis.2007.12.005 PMC2267909

[6] Esau D . Viral causes of lymphoma: the history of epstein‐barr virus and human T‐lymphotropic virus 1. Virology (Auckl). 2017;8:1178122X17731772. 10.1177/1178122X17731772 PMC5621661 28983187

[7] Rho HM , Poiesz B , Ruscetti FW , Gallo RC . Characterization of the reverse transcriptase from a new retrovirus (HTLV) produced by a human cutaneous T‐cell lymphoma cell line. Virology. 1981;112:355‐360. 6166122 10.1016/0042-6822(81)90642-5

[8] Giozza SP , Santos SB , Martinelli M , Porto MA , Muniz AL , Carvalho EM . Salivary and lacrymal gland disorders and HTLV‐1 infection. Rev Stomatol Chir Maxillofac. 2008;109:153‐157. 18374956 10.1016/j.stomax.2007.08.008

[9] Stewart DM , Ramanathan R , Mahanty S , Fedorko DP , Janik JE , Morris JC . Disseminated Strongyloides stercoralis infection in HTLV‐1‐associated adult T‐cell leukemia/lymphoma. Acta Haematol. 2011;126:63‐67. 21474923 10.1159/000324799 PMC3080579

[10] Oliveira PD , Salvino MA , Santos HH , Bittencourt AL . An unusual association of adult T‐cell leukemia/lymphoma with hyalohyphomycosis. Am J Dermatopathol. 2016;38:370‐373. 26981740 10.1097/DAD.0000000000000533

